# The association of human apolipoprotein ε4 with dementia, cognition, imaging, and plasma biomarkers of neurodegeneration in a sample of older adults in the Democratic Republic of the Congo

**DOI:** 10.1002/alz.70735

**Published:** 2025-10-14

**Authors:** Jean Ikanga, Saranya Sundaram Patel, Megan Schwinne, Caterina Alessandra Obenauf, Emmanuel Epenge, Guy Gikelekele, Nathan Tshengele, Immaculee Kavugho, Samuel Mampunza, Lelo Mananga, Charlotte E. Teunissen, Julio C. Rojas, Brandon Chan, Argentina Lario Lago, Adam L. Boxer, Andreas Jeromin, Emile Omba, Alden L. Gross, Alvaro Alonso

**Affiliations:** ^1^ Department of Rehabilitation Medicine Emory University School of Medicine Atlanta Georgia USA; ^2^ School of Medicine Kinshasa, Department of Psychiatry University of Kinshasa and Catholic University of Congo Kinshasa Democratic Republic of Congo; ^3^ OneRehab Dallas Texas USA; ^4^ Department of Biomedical Informatics Emory University School of Medicine Atlanta Georgia USA; ^5^ Department of Neurology University of Kinshasa Kinshasa Democratic Republic of Congo; ^6^ Memory Clinic of Kinshasa Kinshasa Democratic Republic of Congo; ^7^ Amsterdam University Medical Centers Neurochemistry Laboratory Department of Clinical Chemistry Amsterdam Neuroscience, Neurodegeneration Amsterdam University Medical Centers Vrije Universitiet Amsterdam the Netherlands; ^8^ Memory and Aging Center Weill Institute for Neurosciences Department of Neurology University of San Francisco San Francisco California USA; ^9^ ALZpath, Inc. Carlsbad California USA; ^10^ Department of Epidemiology Rollins School of Public Health Emory University Atlanta Georgia USA; ^11^ Department of Epidemiology Johns Hopkins Bloomberg School of Public Health Baltimore Maryland USA

**Keywords:** apolipoprotein E, biomarkers, cognition, Congo, dementia

## Abstract

**INTRODUCTION:**

This study examined the association between the apolipoprotein E (*APOE*) ε4 allele and cognitive performance, neuroimaging, and plasma biomarkers in Congolese older adults in the Democratic Republic of the Congo (DRC).

**METHODS:**

Eighty‐four participants (39 healthy controls [HCs], 45 with suspected dementia), aged 73.0 years on average, were assessed using the African Neuropsychology Battery, magnetic resonance imaging, and blood‐based biomarkers. Regression models adjusted for age, sex, and education evaluated *APOE*’s impact.

**RESULTS:**

*APOE* ε4 was more prevalent in dementia cases than in HCs. Overall, *APOE* ε4 status significantly affected naming and memory scores, mesial temporal and entorhinal cortex atrophy scores, and glial fibrillary acidic protein concentration levels. In HCs, it showed no significant impact on cognitive or neuroimaging tests, except for neurofilament light chain concentration levels. Among dementia participants, *APOE* ε4 status influenced only naming and memory scores.

**DISCUSSION:**

*APOE* ε4 carriers in this DRC cohort showed greater cognitive decline and neurodegeneration, highlighting its significant impact in African populations.

**Highlights:**

- Apolipoprotein E (*APOE*) ε4 was more frequent in dementia cases than in healthy controls in a Democratic Republic of the Congo cohort.
- *APOE* ε4 carriers showed greater cognitive decline, especially in memory and visuospatial skills.
- Neuroimaging findings revealed increased hippocampal atrophy and cortical thinning in carriers.
- Plasma biomarkers in dementia showed higher amyloid beta 40, phosphorylated tau181, neurofilament light chain, and tumor necrosis factor alpha levels.
- Findings underscore *APOE* ε4's impact on neurodegeneration in African populations.

## 1 INTRODUCTION

The human apolipoprotein E (*APOE*) ε4 allele is the strongest genetic risk factor for late‐onset Alzheimer's disease (AD),^1^ and works by promoting the accumulation of amyloid beta (Aβ) peptides, which are a hallmark of AD.^2^, ^3^ *APOE* ε4 also enhances neuroinflammatory responses, alters lipid metabolism,^4^, ^5^, ^6^ and is associated with faster cognitive decline.^7^, ^8^ *APOE* ε4 acts through mechanisms such as Aβ aggregation, tau protein phosphorylation, neurofibrillary tangle formation, synaptic dysfunction, and mitochondrial impairment, all of which contribute to the development of AD.^9^, ^10^

Studies link *APOE* ε4 to cognitive decline, especially in AD patients, with carriers showing greater memory, visuospatial, language, and executive deficits due to tau accumulation and atrophy in the medial temporal, frontal, and parietal lobes.^11^ Some longitudinal studies have indicated that the rate of cognitive decline related to *APOE* ε4 is accelerated in the early stages of the disease.^12^, ^13^ Additional studies have reported an association with regional atrophy in people with dementia, specifically greater hippocampal atrophy in *APOE* ε4 carriers,^14^ as well as temporal lobe atrophy,^15^, ^16^ cortical thinning,^17^, ^18^ and increased white matter lesions.^19^, ^20^ Finally, recent research shows that *APOE* ε4 carriers have higher plasma concentrations of phosphorylated tau (p‐tau)181, neurofilament light chain (NfL), glial fibrillary acidic protein (GFAP) and a lower Aβ42/40 ratio, which are associated with AD and dementia.^21^, ^22^

Most studies have shown that 20% to 30% of the global population carries the *APOE* ε4 variant, while 40% to 60% of individuals with AD have *APOE* ε4.^23^, ^24^ *APOE* ε4/ε4 homozygotes have a 10 to 15 times higher risk of developing AD.^25^, ^26^ The frequency of *APOE* ε4 in cognitively normal European, Black, Hispanic, Asian, and African individuals is 13.7%, 19.0%, 11.0%, 11.0%, and 8.9%, respectively.^25^, ^27^ There is a greater frequency of *APOE* ε4 among individuals with AD across White, Black, Hispanic, Asian, and African identities: 36.7%, 32.2%, 19.2%, 27.8%, and 28.0%, respectively.^25^, ^27^ The odds ratio (OR) for AD when an individual has *APOE* ε4 homozygous among European, Black, Hispanic, and Asian participants is 14.9, 4.7, 2.2, and 33.1, respectively.^25^, ^27^

While *APOE* ε4 is the best‐validated susceptibility genetic variant for late‐onset sporadic AD, its association is weaker in non‐White (e.g., Hispanic, Black, and African) than White ethnic groups.^28^ *APOE* ε4 is more prevalent in Sub‐Saharan African populations, including African Pygmies (40.7%), Tutsi (38.5%), Zairians/Congolese (33.3%), and Fon (29.4%).^29^ However, its association with AD risk is lower in individuals of African descent than in those of European descent (Puerto Ricans: OR = 1.26 vs. 4.49; Black Americans: OR = 2.34 vs. 3.05).^30^ For example, genome‐wide association studies (GWASs) of multiple cohorts of Black Americans have found that the combination of *APOE* ε4 alleles and the gene *ABCA7* increases the risk of AD among Black Americans.^31^ Furthermore, there is a significant difference in the frequency of *APOE* ε4 alleles between normal individuals and those with AD in various ethnic groups worldwide.^24^ The relative increase in the frequency of *APOE* ε4 alleles in AD patients compared to controls is substantially lower in Sub‐Saharan Africa.^32^ The prevalence of the *APOE* ε4 allele among African populations shows considerable variation, ranging from 21.7% among Nigerians to 38.5% among the Tutsi of Burundi, highlighting notable genetic diversity across the continent.

Very few studies have investigated the associations of the *APOE* ε4 allele with cognitive tests, neuroanatomical findings, and neurodegenerative plasma biomarkers in Sub‐Saharan Africa. Hendrie et al. found that among Yoruba/Nigerians, only homozygosity for *APOE* ε4 was a significant risk factor for AD but not for cognitive decline.^27^ Additionally, Ikanga et al. found a significant association between being an *APOE* ε4 carrier and cognitive deficits in Congolese older adults evaluated with the African Neuropsychology Battery (ANB).^33^

Therefore, this preliminary study addresses the gap in Sub‐Saharan Africa research, particularly in French‐speaking countries, by examining *APOE* ε4's association with dementia, cognition, neuroanatomy, and plasma biomarkers in older adults in the Democratic Republic of the Congo (DRC). Based on prior Sub‐Saharan Africa studies, we hypothesized *APOE* ε4 as a significant dementia risk factor linked to cognitive deficits, neuroanatomical lesions, and adverse plasma biomarker levels.

## 2 SUBJECTS/MATERIALS AND METHODS

### 2.1 Study population

**FIGURE 1 alz70735-fig-0001:**
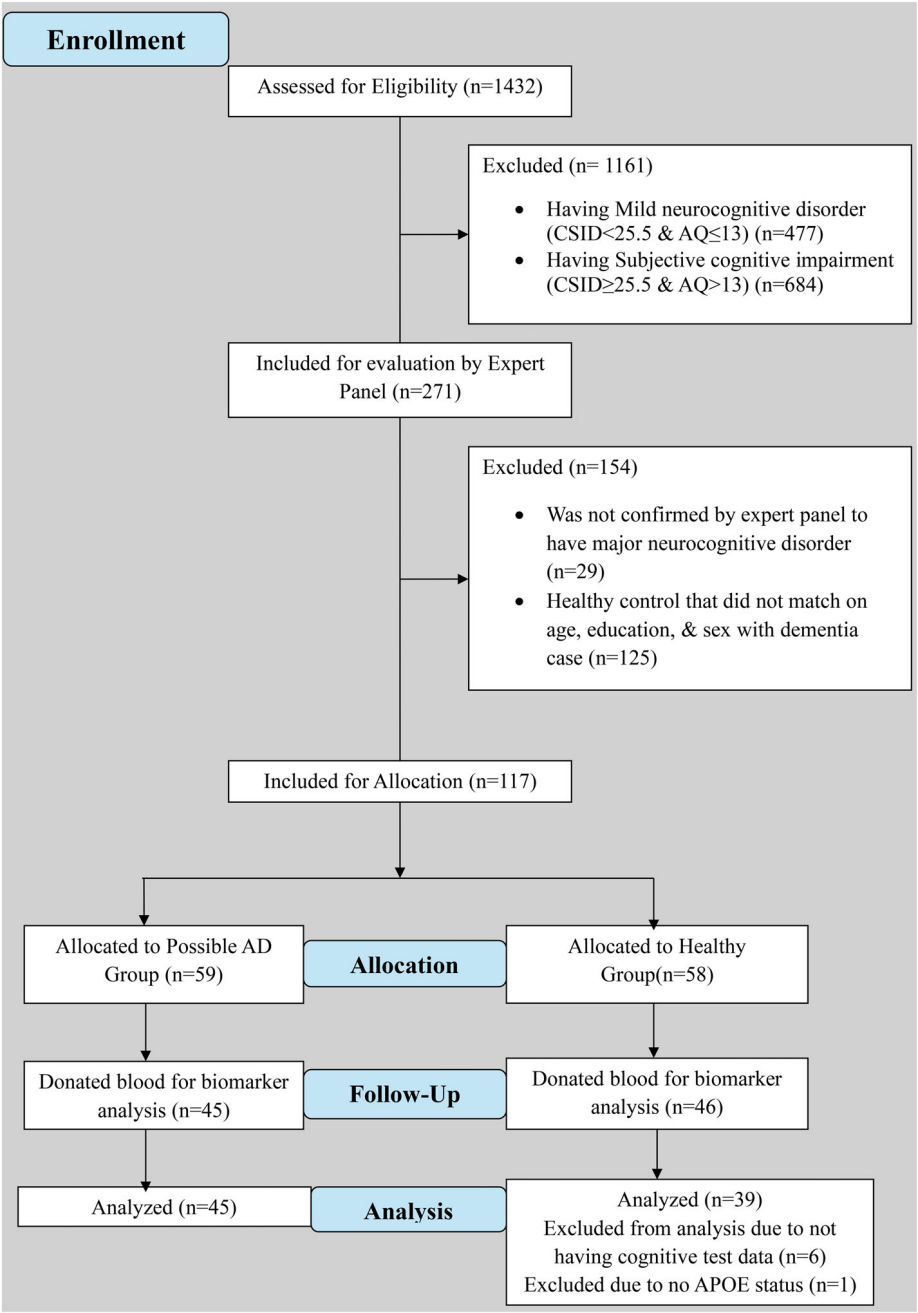
Flow chart of recruitment status from those assessed for eligibility at enrollment (*n* = 1432) to the individuals that were allocated to the possible AD or control group and analyzed (*n* = 84). AD, Alzheimer's disease; *APOE*, apolipoprotein E; AQ, Alzheimer's Questionnaire; CSID, Community Screening Instrument for Dementia.

We screened 1432 Congolese participants for dementia using the Community Screening Instrument for Dementia (CSID)^34^ and the Alzheimer's Questionnaire (AQ),^35^ both widely used in international research. Participants were first categorized based on CSID scores: those scoring < 25.5 were considered cognitively impaired, while scores of ≥ 25.5 indicated cognitive unimpaired status (see Figure 1). Within these two groups, participants were further classified using AQ scores: an AQ score > 13 indicated functional impairment, and a score of ≤ 13 indicated no functional impairment. This process resulted in four subgroups: major neurocognitive disorder/dementia (CSID < 25.5, AQ > 13), mild neurocognitive disorder ([MND]; CSID < 25.5, AQ ≤ 13), subjective cognitive impairment (CSID ≥ 25.5, AQ > 13), and healthy control (HC) with normal cognition (CSID ≥ 25.5, AQ ≤ 13). Only participants classified as having major neurocognitive disorder/dementia or as HC were included in the final study sample.

Out of the initial 1432 participants, 271 individuals met the criteria, including 88 with dementia and 183 HCs. After expert panel review, which included screening tests, clinical interviews, and neurological exams, 59 participants were confirmed to have dementia, while 58 were classified as HCs. Plasma biomarkers were collected from 91 participants (78%), leading to a final sample of 45 individuals with dementia and 46 HCs. Twenty‐six participants declined to provide blood samples. All participants were at least 65 years old, had an informant (family member or friend), and were fluent in either French or Lingala. Exclusion criteria included subjective memory complaints, MND, schizophrenia, and other neurological or medical conditions affecting the central nervous system. Written informed consent was obtained from all participants, who were financially compensated for their time. The study was approved by the ethics committee/institutional review board of the University of Kinshasa.

**RESEARCH IN CONTEXT**

1. **Systematic review**: We conducted a literature search using PubMed and other databases to examine the role of the apolipoprotein E (*APOE*) ε4 allele in cognitive performance, neuroimaging, and plasma biomarkers across diverse populations. While extensive research exists on *APOE* ε4's effects in European and North American cohorts, studies in sub‐Saharan African populations remain limited. Existing research highlights ethnic variability in *APOE* ε4's influence on Alzheimer's disease (AD) risk, but data from Central African populations, particularly in the Democratic Republic of the Congo (DRC), are scarce.
2. **Interpretation**: Our findings demonstrate that *APOE* ε4 carriers in this DRC cohort exhibit increased vulnerability to cognitive decline, neurodegeneration, and biomarker abnormalities, regardless of cognitive status. This supports the hypothesis that *APOE* ε4 has significant effects in African populations, contributing to heightened neurodegenerative processes.
3. **Future directions**: Further studies should investigate gene–environment interactions that may modify *APOE* ε4's effects in African populations. Longitudinal research is needed to determine the allele's predictive value for AD progression. Expanding neuroimaging and biomarker studies in underrepresented regions will enhance our understanding of population‐specific risk factors.

### 2.2 Procedure

Participants[Fig alz70735-fig-0001] were initially screened for dementia using the CSID and AQ measures to assign them to one of four groups. Only those who met Diagnostic and Statistical Manual of Mental Disorders, Fifth Edition criteria for major neurocognitive disorder/dementia or normal cognition underwent further clinical evaluation, including cognitive testing, self‐report questionnaires, and comprehensive psychiatric and neurological assessments to confirm a diagnosis of dementia or classify them as HCs. A diagnostic expert panel, comprising a neurologist (E.E.), psychiatrist (G.G.), and neuropsychologist (J.I.), conducted these assessments.

Participants meeting the criteria for HC or dementia were then interviewed to gather demographic, socioeconomic, and medical history information. During a single session, these participants completed the ANB, as well as questionnaires covering demographic information, medical history, and lifestyle, separate from the diagnostic evaluation. A second visit was scheduled at the Medical Center of Kinshasa (CMK) for blood sample collection, which was performed by a trained phlebotomist, and for neuroimaging. After each session, participants received appropriate compensation based on their level of participation.

### 2.3 Measures

#### 2.3.1 Cognitive measures

Cognitive function was evaluated using the ANB,^36^ which includes the: African Naming Test ([ANT]; confrontation naming; total unaided correct score), African List Memory Test ([ALMT]; verbal learning and memory; total long delay free recall correct score), African Visuospatial Memory Test ([AVMT]; visuospatial memory; total long delay recall correct score), African Proverb Test ([APT]; abstract reasoning; total interpretation correct score), and African Card Game Test ([ACGT]; problem solving; total wins correct score). The ANB has demonstrated good psychometric properties in evaluating effects of aging and neurological disease alongside providing culturally and linguistically appropriate neuropsychological measures for Sub‐Saharan African countries.^36^

#### 2.3.2 Neuroimaging

All participants were imaged on a 1.5 Tesla magnetic resonance imaging unit (Siemens, Magneton Sonata) scanner at HJ Hospitals in Kinshasa using the same standardized imaging acquisition protocol based on the Alzheimer's Disease Research Center protocol of Emory University. This consisted of sagittal volumetric T1‐weighted (magnetization‐prepared rapid gradient echo [MPRAGE]), coronal T2‐weighted, and axial diffusion‐weighted, T2‐weighted, and T2 fluid‐attenuated inversion recovery sequences. Typical acquisition parameters for the MPRAGE sequence were repetition time = 2200 ms, minimum full echo time, inversion time = 1000 ms, flip angle = 8°, and field of view = 25 cm, with a 192 × 184 acquisition matrix, yielding a voxel size of ≈ 1.25 × 1.25 × 1.2 mm.^37^ Images were reviewed by an experienced neuroradiologist. White matter hyperintensity was graded according to the age‐related white matter changes (ARWMC) scale.^38^ Number of chronic brain parenchymal microhemorrhages were recorded. The lobar volume loss pattern of the brain was assessed. MPRAGE images were reoriented into the oblique coronal plane orthogonal to the principal axis of the hippocampal formation, and medial temporal lobe atrophy (MTLA) and entorhinal cortex atrophy (EriCa) scores were assessed.^39^ Finally, the presence or absence of any additional abnormalities was noted, and patients were excluded if neuroimaging evidence indicated an etiology other than probable AD (e.g., presence of a brain tumor). In neuroimaging data interpretation, a higher volume of a structure is generally considered beneficial, whereas atrophy indicates pathology. Regarding biomarkers, a low Aβ42/40 ratio is pathological. Additionally, elevated concentration levels of other plasma biomarkers are also indicative of pathology.

The 3D T1w images were segmented using FreeSurfer (v.6), which includes a full processing stream for magnetic resonance imaging data that involves skull‐stripping, bias field correction, registration, and anatomical segmentation, as well as cortical surface reconstruction, registration, and parcellation. Regional brain volume for both cortical and subcortical brain regions was calculated. The left and right hippocampal (LH, RH) volumes were averaged. Interindividual variation in head size was accounted for in further statistical analysis by controlling for total intracranial volume.

Blood samples were drawn in the CMK blood laboratory by venipuncture into dipotassium ethylene diamine tetra acetic acid (K_2_ EDTA) tubes. Samples were centrifuged within 15 minutes, and 5 mL of plasma was aliquoted into separate 0.5 mL polypropylene tubes and stored initially at −20°C for less than a week and then moved to a −80°C freezer for longer term storage at a CMK laboratory.^40^ These aliquots were shipped frozen on dry ice to Emory University. Plasma biomarker concentrations were measured using commercially available Neurology 4‐PLEX E (Aβ40, Aβ42, NfL, and GFAP; lot #503819), P‐Tau181 (P‐Tau181 v2; lot #503732), IL‐1b (lot #503806) and IL‐10 (IL‐10 2.0, lot #503533) Quanterix kits on the Simoa HD‐X platform (Billerica, MA) at UCSF.^41^, ^42^, ^43^ The *APOE* isoform‐specific peptides (ε2, ε3, and ε4) were detected and identified using liquid chromatography tandem mass spectrometry by C2N diagnostics, which have been previously shown to be 100% concordant with genotype.^41^

#### 2.3.3 Statistical analyses

Statistical analyses were conducted using R version 4. Participants with missing *APOE* genotype data or incomplete neurological status classification (HC vs. suspected dementia) were excluded. Descriptive statistics were used to summarize the data, with continuous variables reported as means with standard deviations (SDs) and categorical variables as frequencies with column percentages. To compare clinical variables—including demographics, screening and cognitive test results, *APOE* genotype, neuroimaging findings, and plasma biomarkers—by neurological status, linear regression models were used for continuous variables, while logistic regression was applied to categorical variables. Age, sex, Geriatric Depression Scale (GDS) score, and education were controlled for in the analysis. Age was included to account for its impact on clinical and biological measures, while sex was adjusted for due to known differences in cognitive performance and biomarkers. GDS score was included to mitigate the confounding effect of depression on cognition and neuroimaging, and education was considered to control for cognitive reserve, which can influence outcomes and biomarkers. For analyses involving neuroimaging data, total intracranial volume was also used as a covariate, as it adjusts for individual differences in head size. Clinical variables were further analyzed based on *APOE* ε4 genotype (ε4 allele carriers vs. non‐carriers) across the entire cohort, as well as separately for cognitively healthy individuals and those with suspected dementia.

Clinical variables were further analyzed based on *APOE* ε4 genotype (ε4 allele carriers vs. non‐carriers) across the entire cohort, as well as separately for cognitively healthy individuals and those with suspected dementia. The same statistical techniques were applied, and Cohen *d* was included to quantify the effect size between *APOE* ε4 groups, with values of ≥ 0.80 indicating a large effect size. All results were reported with 95% confidence intervals, and statistical significance was set at *p* < 0.05.

## 3 RESULTS

Demographic data, cognitive scores, clinical data, and plasma biomarker concentrations, stratified by neurological status, are presented in Table 1. The sample consisted of 84 participants, of whom 46 (55.4%) were female. The average age was 73.0 years (SD = 7.7 years), and the participants had an average of 8.5 years of education (SD = 5.3 years). As expected, there were significant differences in cognitive screening scores used to distinguish neurological status, with healthy individuals scoring better than those with dementia. For clinical data, there were significant statistical differences between groups with the ε3/ε3 genotype and ε4 carriers. The prevalence of the ε3/ε3 genotype was higher in the HC group (53.8%, *p* = 0.03), while the prevalence of ε4 carriers was higher in the dementia group (64.4% vs. HC = 33.3%, *p* = 0.004). Neuroimaging analyses showed significant atrophy in the mesial temporal and entorhinal cortex regions in the dementia group compared to the HC group. Plasma biomarker data showed differences in GFAP and NfL concentrations, with higher levels in the suspected dementia group.

**TABLE 1:** Characteristics of the sample stratified by neurological status.

Variables, mean (SD)	All (*n* = 84)	Healthy controls (*n* = 39)	Suspected dementia (*n* = 45)	*p* value
**Demographics**				
Age	73.0 (7.7)	72.0 (7.9)	73.8 (7.6)	0.33
Female^a^	46 (55.4%)	21 (55.2%)	25 (55.6%)	0.64
Education	8.5 (5.3)	9.6 (4.9)	7.4 (5.5)	0.31
**Screening and cognitive tests**				
CSID	24.9 (7.6)	31.9 (2.8)	19.6 (5.6)	<0.0001
AQ	12.1 (8.8)	3.2 (2.7)	19.3 (4.0)	<0.0001
ANT	18.5 (6.7)	21.7 (4.1)	15.5 (7.2)	0.0008
ALMT Trial 1	4.0 (2.2)	5.5 (1.6)	2.6 (1.7)	<0.0001
ALMT Trial 3	5.8 (2.7)	7.8 (2.0)	4.0 (1.9)	<0.0001
ALMT Recall	3.3 (3.4)	6.7 (1.6)	0.31 (0.64)	<0.0001
AVMT Trial 1	2.7 (2.7)	4.2 (2.9)	1.3 (1.6)	0.0001
AVMT Trial 3	5.0 (4.7)	8.5 (4.5)	1.9 (1.9)	<0.0001
AVMT Recall	4.3 (4.7)	7.8 (4.3)	1.0 (1.7)	<0.0001
Proverb Test	4.6 (4.4)	6.9 (5.1)	2.5 (2.2)	<0.0001
African Card Game Wins	24.8 (9.0)	28.2 (9.8)	21.7 (7.0)	0.077
GDS	5.8 (3.5)	3.8 (2.4)	7.5 (3.5)	<0.0001
***APOE* genetics**				
ε4/ε4^a^	8 (9.5%)	0 (0)	8 (17.8%)	0.99
ε3/ε4^a^	32 (38.1%)	11 (28.2%)	21 (46.7%)	0.060
ε3/ε3^a^	35 (41.7%)	21 (53.8%)	14 (31.1%)	0.17
ε2/ε3^a^	7 (8.3%)	5 (12.8%)	2 (4.4%)	0.36
ε2/ε4^a^	2 (2.4%)	2 (5.1%)	0 (0%)	0.98
ε4 carrier^a^	42 (50%)	13 (33.3%)	29 (64.4%)	0.017
**Neuroimaging** ^b^				
Intracranial volume	1434439 (232268)	1435661 (142774)	1433637 (277941)	0.47
Left hippocampal volume	3154 (553)	3434 (463)	2970 (535)	0.073
Right hippocampal volume	3138 (558)	3388 (435)	2973 (573)	0.092
Left entorhinal cortex volume	1641 (512)	1818 (341)	1525 (573)	0.29
Right entorhinal cortex volume	1742 (551)	1895 (496)	1641 (568)	0.52
Microhemorrhage^a^	15 (30.6%)	6 (26.1%)	9 (34.6%)	0.43
White matter hyperintensity	70.2 (3.1)	70.4 (3.8)	70.0 (2.6)	0.98
Mesial temporal atrophy score	1.6 (1.3)	0.58 (0.78)	2.3 (1.1)	<0.0001
Entorhinal cortex atrophy score	1.1 (0.9)	0.33 (0.48)	1.68 (0.78)	<0.0001
**Plasma biomarkers** ^b^				
Aβ_42_ (pg/mL)	3.8 (2.1)	3.9 (2.3)	3.8 (2.0)	0.37
Aβ_40_ (pg/mL)	74.4 (50.8)	69.7 (51.9)	78.4 (50.1)	0.75
Aβ_42/40_	0.069 (0.035)	0.077 (0.040)	0.062 (0.031)	0.17
p‐tau_181_	2.7 (2.0)	2.3 (1.5)	3.0 (2.3)	0.42
Aβ_42_/p‐tau_181_	4.8 (16.7)	24.5 (46.7)	4.5 (49.7)	0.42
NfL	51.0 (39.3)	37.5 (32.2)	62.7 (41.5)	0.14
GFAP	208 (130)	169 (100)	241 (144)	0.22
TNF‐α	0.60 (0.28)	0.62 (0.34)	0.58 (0.23)	0.96
IL‐6	0.012 (0.013)	0.011 (0.012)	0.013 (0.014)	0.86
IL‐10	0.31 (0.36)	0.35 (0.37)	0.27 (0.34)	0.75

*Note*: All individuals in this table have *APOE* phenotype data. Statistical test uses linear regression, controlling for age, sex, GDS, and education for all. Neuroimaging measures additionally controls for intracranial volume. Descriptive variables are the outcome.

Abbreviations: Aβ, amyloid beta; ALMT, African List Memory Test; ANT, Arican Naming Test; *APOE*, apolipoprotein; AQ, Alzheimer's Questionnaire; AVMT, African Visuospatial Memory Test; CI, confidence interval; CSID, Community Screening Instrument for Dementia; GDS, Geriatric Depression Scale; GFAP, glial fibrillary acidic protein; IL, interleukin; NfL, neurofilament light chain; p‐tau, phosphorylated tau; SD, standard deviation; TNF‐α, tumor necrosis factor alpha.

^a^ Results presented as *n* (%) with logistic regression for statistical test.

^b^ In neuroimaging data interpretation, a higher volume of a structure is generally considered beneficial, whereas atrophy indicates pathology. Regarding biomarkers, a low Aβ42/40 ratio is pathological. Additionally, elevated concentration levels of other plasma biomarkers are also indicative of pathology.

**FIGURE 2:**
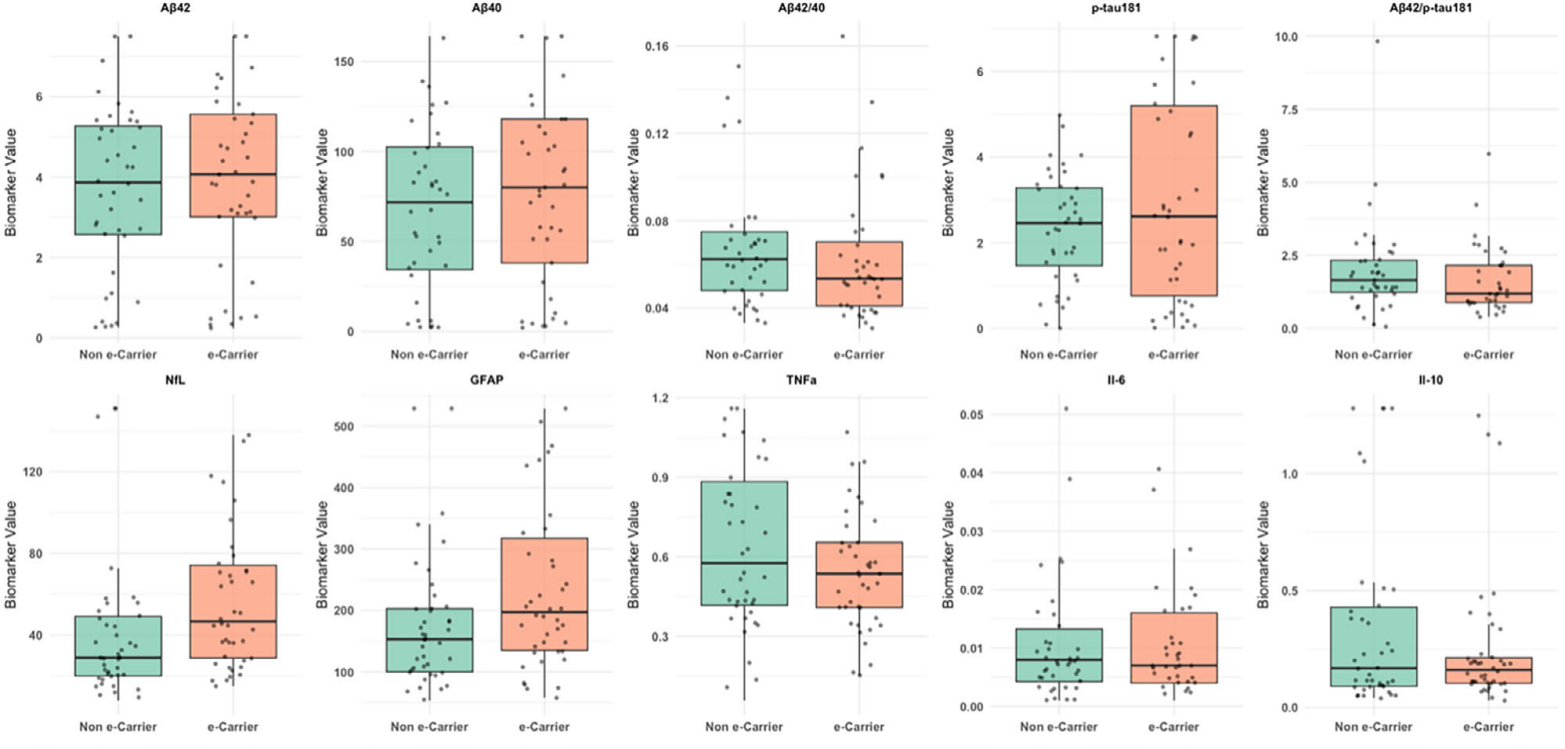
Boxplots of biomarker values stratified by *APOE* ε4 carrier status. Boxplots display distributions of biomarker values stratified by *APOE* ε4 carrier status (non ε‐carrier vs. ε‐carrier). Biomarkers include Aβ42, Aβ40, Aβ42/40, p‐tau181, Aβ42/p‐tau181, NfL, GFAP, TNF‐α, IL‐6, and IL‐10. Each box represents the IQR, with the median shown as a horizontal line and whiskers extending to 1.5 × IQR. Individual data points are overlaid. These comparisons illustrate potential group differences in plasma‐based biomarkers of Alzheimer's disease and neuroinflammation. Aβ, amyloid beta; *APOE*, apolipoprotein; GFAP, glial fibrillary acidic protein; IL, interleukin; IQR, interquartile range; p‐tau, phosphorylated tau; TNF‐α, tumor necrosis factor alpha.

Figure 2 presents the box plot distribution of biomarker values stratified by *APOE* ε4 carrier status.

**FIGURE 3:**
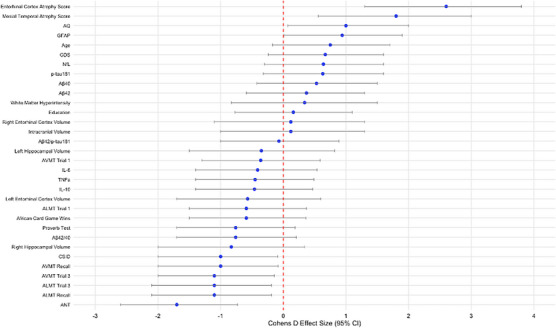
Forest plot of Cohen *d* effect sizes for *APOE* genotype comparisons in the full sample (*n* = 84). Cohen *d* effect sizes (± 95% confidence intervals) for group comparisons based on *APOE* ε4 carrier status across clinical, cognitive, biomarker, and neuroimaging variables in the full sample (*n* = 84). Models were adjusted for age, sex, years of education, and GDS score; neuroimaging measures were additionally adjusted for intracranial volume. Positive values reflect higher scores in APOE ε4 carriers; negative values reflect higher scores in non‐carriers. Key biomarkers and neurodegeneration indicators (e.g., GFAP, Aβ42/p‐tau181 ratio, entorhinal cortex atrophy) show strong associations with APOE genotype. Aβ, amyloid beta; ALMT, African List Memory Test; ANT, Arican Naming Test; *APOE*, apolipoprotein; AVMT, African Visuospatial Memory Test; CI, confidence interval; CSID, Community Screening Instrument for Dementia; GDS, Geriatric Depression Scale; GFAP, glial fibrillary acidic protein; IL, interleukin; NfL, neurofilament light chain; p‐tau, phosphorylated tau; TNF‐α, tumor necrosis factor alpha.

Table 2 and Figure 3 present Cohen *d* effect size, quantifying the difference between ε4 carriers and non‐carriers across all participants, including both HCs and individuals suspected of dementia. Cognitively, there were significant strong relationships between CSID, AQ, ANT, ALMT 1, 3, recall, AVMT 3, and recall scores, and the presence of the *APOE* ε4 allele. Neuroimaging results revealed significant large effect sizes of *APOE* ε4 in several measures, including MTLA and EriCa scores. Plasma biomarker analyses indicated a significant strong relationship of the *APOE* ε4 allele with GFAP concentration levels.

**TABLE 2:** Comparisons of all participants (*n* = 84) based on *APOE* ε4 genotypes.

Variables, mean (SD)	Non‐carriers (*n* = 42)	ε4 carriers (*n* = 42)	Effect size (95% CI)	*p* value
**Demographics**				
Age	71.8 (7.1)	74.1 (8.2)	0.75 (−0.17, 1.7)	0.11
Female^a^	26 (61.9%)	20 (48.8%)	0.64 (0.20, 2.1)	0.45
Education	8.4 (5.4)	8.5 (5.3)	0.16 (−0.77, 1.1)	0.74
**Screening and cognitive tests**				
CSID	26.9 (7.9)	23.0 (6.9)	−1.0 (−2.0, −0.09)	0.036
AQ	9.7 (8.5)	14.6 (8.5)	1.0 (0.07, 2.0)	0.038
ANT	21.0 (4.8)	15.8 (7.4)	−1.7 (−2.6, −0.73)	0.0009
ALMT Trial 1	4.4 (2.1)	3.5 (2.2)	−0.59 (−1.5, 0.37)	0.23
ALMT Trial 3	6.6 (2.7)	4.9 (2.5)	−1.1 (−2.1, −0.19)	0.021
ALMT Recall	4.4 (3.4)	2.2 (3.0)	−1.1 (−2.1, −0.19)	0.022
AVMT Trial 1	3.1 (2.8)	2.2 (2.6)	−0.36 (−1.3, 0.59)	0.46
AVMT Trial 3	6.3 (4.7)	3.7 (4.4)	−1.1 (−2.0, −0.14)	0.027
AVMT Recall	5.5 (4.5)	3.0 (4.5)	−1.0 (−2.0, −0.08)	0.036
Proverb Test	5.4 (5.1)	3.7 (3.4)	−0.76 (−1.7, 0.19)	0.12
African Card Game Wins	26.2 (9.7)	23.3 (8.1)	−0.59 (−1.5, 0.36)	0.23
GDS	5.3 (3.7)	6.3 (3.3)	0.67 (−0.24, 1.6)	0.15
**Neuroimaging** ^b^				
Intracranial volume	1401957 (156288)	1469241 (291938)	0.12 (−1.0, 1.3)	0.84
Left hippocampal volume	3199 (493)	3106 (616)	−0.35 (−1.5, 0.82)	0.56
Right hippocampal volume	3238 (478)	3031 (623)	−0.83 (−2.0, 0.34)	0.17
Left entorhinal cortex volume	1677 (517)	1603 (513)	−0.57 (−1.7, 0.60)	0.35
Right entorhinal cortex volume	1717 (523)	1769 (587)	0.12 (−1.1, 1.3)	0.84
Microhemorrhage^a^	6 (24.0%)	9 (37.5%)	1.7 (0.41, 6.9)	0.33
White matter hyperintensity	69.9 (3.5)	70.5 (2.7)	0.34 (−0.83, 1.5)	0.57
Mesial temporal atrophy score	1.1 (1.3)	2.1 (1.1)	1.8 (0.56, 3.0)	0.006
Entorhinal cortex atrophy score	0.73 (0.83)	1.6 (0.89)	2.6 (1.3, 3.8)	0.0001
**Plasma biomarkers** ^b^				
Aβ_42_	3.7 (2.1)	4.0 (2.2)	0.37 (−0.59, 1.3)	0.46
Aβ_40_	68.6 (47.8)	80.0 (53.6)	0.53 (−0.42, 1.5)	0.28
Aβ_42/40_	0.073 (0.038)	0.064 (0.033)	−0.76 (−1.7, 0.21)	0.13
p‐tau_181_	2.4 (1.3)	3.0 (2.4)	0.63 (−0.32, 1.6)	0.19
Aβ_42_/p‐tau_181_	5.5 (22.1)	4.2 (9.1)	−0.07 (−1.0, 0.89)	0.88
NfL	42.3 (38.6)	59.7 (38.5)	0.64 (−0.30, 1.6)	0.18
GFAP	175 (110)	240 (141)	0.94 (0.01, 1.9)	0.052
TNF‐α	0.64 (0.32)	0.55 (0.24)	−0.45 (−1.4, 0.49)	0.35
IL‐6	0.012 (0.013)	0.012 (0.013)	−0.41 (−1.4, 0.54)	0.40
IL‐10	0.36 (0.41)	0.25 (0.28)	−0.46 (−1.4, 0.47)	0.33

*Note*: Statistical test uses linear regression, controlling for age, sex, GDS, and education for all. Neuroimaging measures additionally controls for intracranial volume. Descriptive variables are the outcome. The effect size measure is Cohen *d* (except for in the categorical outcomes: sex and microhemorrhages).

Abbreviations: Aβ, amyloid beta; ALMT, African List Memory Test; ANT, Arican Naming Test; *APOE*, apolipoprotein; AQ, Alzheimer's Questionnaire; AVMT, African Visuospatial Memory Test; CI, confidence interval; CSID, Community Screening Instrument for Dementia; GDS, Geriatric Depression Scale; GFAP, glial fibrillary acidic protein; IL, interleukin; NfL, neurofilament light chain; p‐tau, phosphorylated tau; SD, standard deviation; TNF‐α, tumor necrosis factor alpha.

^a^ Results presented as *n* (%) and odds ratio is presented for the effect size measure; logistic regression is used for the statistical test.

^b^ In neuroimaging data interpretation, a higher volume of a structure is generally considered beneficial, whereas atrophy indicates pathology. Regarding biomarkers, a low Aβ42/40 ratio is pathological. Additionally, elevated concentration levels of other plasma biomarkers are also indicative of pathology.

**FIGURE 4 alz70735-fig-0004:**
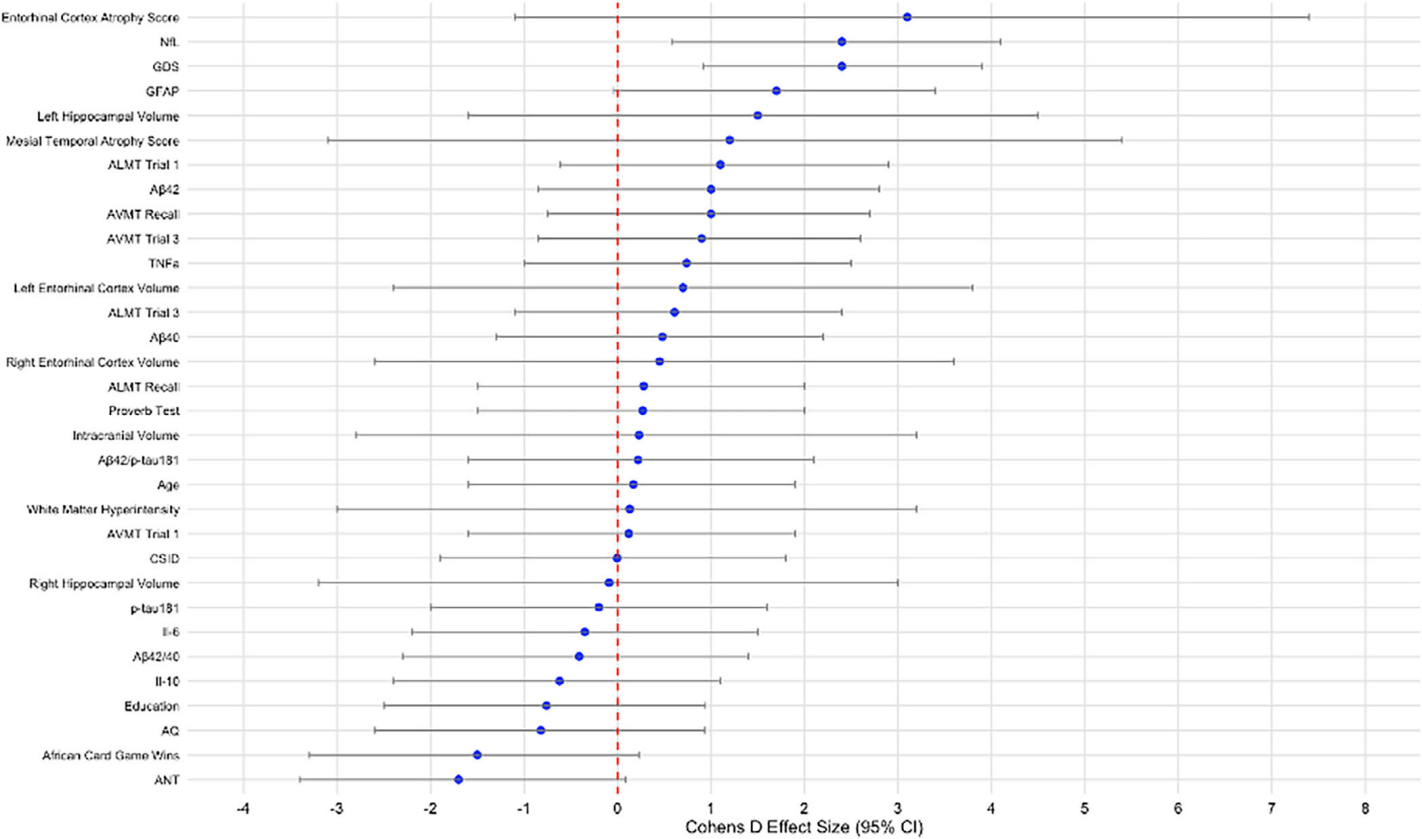
Forest plot of Cohen *d* effect sizes for *APOE* genotype comparisons in healthy controls (*n* = 39). Cohen *d* effect sizes (± 95% confidence intervals) for group comparisons based on *APOE* ε4 carrier status restricted to healthy control participants (*n* = 39). Models were adjusted for age, sex, years of education, and GDS score; neuroimaging measures were additionally adjusted for intracranial volume. This analysis isolates genotype effects in cognitively unimpaired individuals. Notable differences include elevated GFAP and NfL levels and greater entorhinal atrophy among ε carriers, suggesting early biomarker alterations associated with genetic risk. Aβ, amyloid beta; ALMT, African List Memory Test; ANT, Arican Naming Test; *APOE*, apolipoprotein; AVMT, African Visuospatial Memory Test; CI, confidence interval; CSID, Community Screening Instrument for Dementia; GDS, Geriatric Depression Scale; GFAP, glial fibrillary acidic protein; IL, interleukin; NfL, neurofilament light chain; p‐tau, phosphorylated tau; TNF‐α, tumor necrosis factor alpha.

Table 3 and Figure 4 present the Cohen *d* effect size quantifying the difference between ε4 carriers and non‐carriers among HCs. Cognitive analyses did not show a strong relationship of *APOE* ε4 carrier status with cognitive scores. Additionally, neuroimaging results did not show any significant large effect of *APOE* ε4 carriers on neuroimaging data. Plasma biomarker findings indicated that *APOE* ε4 status was strongly and significantly associated with NfL concentrations.

**TABLE 3 alz70735-tbl-0003:** Comparisons of healthy controls (*n =* 39) based on *APOE* ε4 genotypes.

Variables, mean (SD)	Non‐carriers (*n* = 26)	ε4 carriers (*n* = 13)	Effect size (95% CI)	*p* value
**Demographics**				
Age	71.2 (7.4)	73.6 (9.0)	0.17 (−1.6, 1.9)	0.85
Female^a^	14 (53.8%)	7 (58.3%)	0.95 (0.13, 6.8)	0.96
Education	10.1 (4.9)	8.6 (5.0)	−0.76 (−2.5, 0.94)	0.39
**Screening and cognitive tests**				
CSID	32.1 (2.5)	31.5 (3.4)	−0.005 (−1.9, 1.8)	0.99
AQ	3.3 (2.6)	3.0 (2.9)	−0.82 (−2.6, 0.93)	0.37
ANT	22.7 (3.3)	19.8 (5.0)	−1.7 (−3.4, 0.09)	0.072
ALMT Trial 1	5.4 (1.6)	5.7 (1.7)	1.1 (−0.61, 2.9)	0.21
ALMT Trial 3	7.9 (2.0)	7.5 (1.9)	0.61 (−1.1, 2.4)	0.50
ALMT Recall	6.8 (1.7)	6.4 (1.4)	0.28 (−1.5, 2.0)	0.75
AVMT Trial 1	4.5 (2.6)	3.6 (3.5)	0.12 (−1.6, 1.9)	0.89
AVMT Trial 3	8.9 (3.8)	7.6 (5.8)	0.90 (−0.85, 2.6)	0.32
AVMT Recall	8.0 (3.6)	7.4 (5.7)	1.0 (−0.75, 2.7)	0.27
Proverb Test	7.4 (5.4)	6.0 (4.4)	0.27 (−1.5, 2.0)	0.76
African Card Game Wins	29.4 (9.5)	25.7 (10.4)	−1.5 (−3.3, 0.23)	0.098
GDS	3.0 (1.8)	5.3 (2.7)	2.4 (0.92, 3.9)	0.003
**Neuroimaging** ^b^				
Intracranial volume	1424874 (151445)	1474492 (110586)	0.23 (−2.8, 3.2)	0.88
Left hippocampal volume	3331 (393)	3805 (550)	1.5 (−1.6, 4.5)	0.37
Right hippocampal volume	3330 (440)	3596 (387)	−0.09 (−3.2, 3.0)	0.95
Left entorhinal cortex volume	1787 (372)	1930 (181)	0.70 (−2.4, 3.8)	0.66
Right entorhinal cortex volume	1848 (462)	2066 (633)	0.45 (−2.6, 3.6)	0.78
Microhemorrhage^a^	4 (22.2%)	2 (40.0%)	2.3 (0.28, 19.2)	0.67
White matter hyperintensity	70.4 (4.0)	70.6 (3.5)	0.13 (−3.0, 3.2)	0.94
Mesial temporal atrophy score	0.44 (0.78)	1.0 (0.63)	1.2 (−3.1, 5.4)	0.60
Entorhinal cortex atrophy score	0.28 (0.46)	0.50 (0.55)	3.1 (−1.1, 7.4)	0.17
**Plasma biomarkers** ^b^				
Aβ_42_	3.6 (2.0)	4.5 (2.8)	1.0 (−0.85, 2.8)	0.30
Aβ_40_	64.2 (46.0)	79.9 (62.1)	0.48 (−1.3, 2.2)	0.60
Aβ_42/40_	0.077 (0.038)	0.076 (0.044)	−0.41 (−2.3, 1.4)	0.66
p‐tau_181_	2.3 (1.2)	2.2 (2.0)	−0.20 (−2.0, 1.6)	0.82
Aβ_42_/p‐tau_181_	7.6 (28.5)	7.4 (14.5)	0.22 (−1.6, 2.1)	0.82
NfL	30.5 (27.7)	51.6 (36.8)	2.4 (0.58, 4.1)	0.014
GFAP	145 (70.3)	217 (133)	1.7 (−0.04, 3.4)	0.065
TNF‐α	0.63 (0.35)	0.60 (0.32)	0.74 (−1.0, 2.5)	0.41
IL‐6	0.011 (0.012)	0.011 (0.011)	−0.35 (−2.2, 1.5)	0.71
IL‐10	0.36 (0.41)	0.33 (0.31)	−0.62 (−2.4, 1.1)	0.49

*Note*: Statistical test uses linear regression, controlling for age, sex, GDS, and education for all. Neuroimaging measures additionally controls for intracranial volume. Descriptive variables are the outcome. The effect size measure is Cohen *d* (except for in the categorical outcomes: sex and microhemorrhages).

Abbreviations: Aβ, amyloid beta; ALMT, African List Memory Test; ANT, Arican Naming Test; *APOE*, apolipoprotein; AQ, Alzheimer's Questionnaire; AVMT, African Visuospatial Memory Test; CI, confidence interval; CSID, Community Screening Instrument for Dementia; GDS, Geriatric Depression Scale; GFAP, glial fibrillary acidic protein; IL, interleukin; NfL, neurofilament light chain; p‐tau, phosphorylated tau; SD, standard deviation; TNF‐α, tumor necrosis factor alpha.

^a^ Results presented as *n* (%) and odds ratio is presented for the effect size measure; logistic regression is used for the statistical test.

^b^ In neuroimaging data interpretation, a higher volume of a structure is generally considered beneficial, whereas atrophy indicates pathology. Regarding biomarkers, a low Aβ42/40 ratio is pathological. Additionally, elevated concentration levels of other plasma biomarkers are also indicative of pathology.

**FIGURE 5 alz70735-fig-0005:**
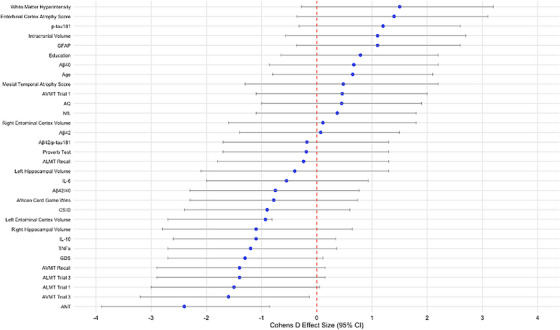
Forest plot of Cohen *d* effect sizes for *APOE* genotype comparisons in participants with suspected dementia (*n* = 45). Cohen *d* effect sizes (± 95% confidence intervals) for group comparisons based on APOE ε4 carrier status in participants with suspected dementia (*n* = 45). Models were adjusted for age, sex, years of education, and GDS score; neuroimaging measures were additionally adjusted for intracranial volume. Positive values reflect higher scores in *APOE* ε4 carriers; negative values reflect higher scores in non‐carriers. Findings reveal prominent genotype‐related differences in markers of neurodegeneration (e.g., white matter hyperintensities, entorhinal cortex atrophy), biomarkers (e.g., p‐tau181, GFAP), and select cognitive outcomes, underscoring the influence of *APOE* status in symptomatic individuals. Aβ, amyloid beta; ALMT, African List Memory Test; ANT, Arican Naming Test; *APOE*, apolipoprotein; AVMT, African Visuospatial Memory Test; CI, confidence interval; CSID, Community Screening Instrument for Dementia; GDS, Geriatric Depression Scale; GFAP, glial fibrillary acidic protein; IL, interleukin; NfL, neurofilament light chain; p‐tau, phosphorylated tau; TNF‐α, tumor necrosis factor alpha.

Table 4 and Figure 5 present Cohen *d* effect size, quantifying the difference between ε4 carriers and non‐carriers among individuals with suspected dementia. Cognitive analyses revealed significant large effect sizes of the *APOE* ε4 allele on ANT and AVMT 3 scores. Neuroimaging results and plasma biomarker findings did not show any significant strong relationship with *APOE* ε4 status.

**TABLE 4 alz70735-tbl-0004:** Comparisons of individuals with suspected dementia (*n = 45*) based on *APOE* ε4 genotypes.

Variables, mean (SD)	Non‐carriers (*n* = 16)	ε4 carriers (*n* = 29)	Effect size (95% CI)	*p* value
**Demographics**				
Age	72.9 (6.7)	74.3 (8.0)	0.65 (−0.80, 2.1)	0.38
Female^a^	12 (75.0%)	13 (44.8%)	0.39 (0.06, 2.5)	0.32
Education	5.5 (5.2)	8.5 (5.5)	0.79 (−0.65, 2.2)	0.29
**Screening and cognitive tests**				
CSID	19.4 (6.9)	19.8 (4.8)	−0.90 (−2.4, 0.60)	0.24
AQ	19.3 (3.6)	19.3 (4.3)	0.45 (−1.0, 1.9)	0.55
ANT	18.0 (5.7)	14.1 (7.6)	−2.4 (−3.9, −0.85)	0.004
ALMT Trial 1	2.8 (1.7)	2.5 (1.7)	−1.5 (−3.0, 0.05)	0.067
ALMT Trial 3	4.3 (2.2)	3.8 (1.7)	−1.4 (−2.9, 0.15)	0.085
ALMT Recall	0.33 (0.62)	0.30 (0.67)	−0.24 (−1.8, 1.3)	0.76
AVMT Trial 1	0.73 (0.80)	1.6 (1.8)	0.46 (−1.1, 2.0)	0.56
AVMT Trial 3	1.8 (2.0)	1.9 (1.9)	−1.6 (−3.2, −0.13)	0.041
AVMT Recall	1.1 (1.9)	0.96 (1.7)	−1.4 (−2.9, 0.15)	0.086
Proverb Test	2.1 (2.0)	2.7 (2.3)	−0.19 (−1.7, 1.3)	0.81
African Card Game Wins	20.8 (7.4)	22.3 (6.8)	−0.78 (−2.3, 0.74)	0.32
GDS	8.8 (3.0)	6.7 (3.5)	−1.3 (−2.7, 0.11)	0.079
**Neuroimaging** ^b^				
Intracranial volume	1367582 (163711)	1468100 (319947)	1.1 (−0.57, 2.7)	0.21
Left hippocampal volume	3002 (576)	2954 (526)	−0.40 (−2.1, 1.3)	0.65
Right hippocampal volume	3099 (516)	2907 (601)	−1.1 (−2.8, 0.64)	0.23
Left entorhinal cortex volume	1513 (664)	1531 (536)	−0.93 (−2.7, −0.81)	0.30
Right entorhinal cortex volume	1520 (566)	1705 (571)	0.11 (−1.6, 1.8)	0.90
Microhemorrhage^a^	2 (28.6%)	7 (36.8%)	1.2 (0.12, 12.4)	0.87
White matter hyperintensity	69.2 (2.6)	70.5 (2.6)	1.5 (−0.28, 3.2)	0.11
Mesial temporal atrophy score	2.1 (1.2)	2.4 (1.0)	0.48 (−1.3, 2.2)	0.60
Entorhinal cortex atrophy score	1.4 (0.79)	1.8 (0.76)	1.4 (−0.36, 3.1)	0.13
**Plasma biomarkers** ^b^				
Aβ_42_	3.9 (2.2)	3.8 (2.0)	0.07 (−1.4, 1.5)	0.93
Aβ_40_	75.3 (51.1)	80.1 (50.3)	0.67 (−0.86, 2.2)	0.40
Aβ_42/40_	0.067 (0.037)	0.059 (0.026)	−0.75 (−2.3, 0.77)	0.34
p‐tau_181_	2.4 (1.4)	3.4 (2.6)	1.2 (−0.32, 2.6)	0.13
Aβ_42_/p‐tau_181_	2.3 (2.3)	2.83 (5.4)	−0.18 (−1.7, 1.3)	0.81
NfL	61.5 (46.5)	63.4 (39.3)	0.37 (−1.1, 1.8)	0.63
GFAP	224 (143)	250 (146)	1.1 (−0.37, 2.6)	0.15
TNF‐α	0.67 (0.28)	0.54 (0.19)	−1.2 (−2.7, 0.36)	0.14
IL‐6	0.014 (0.016)	0.012 (0.014)	−0.55 (−2.0, 0.93)	0.47
IL‐10	0.37 (0.44)	0.21 (0.27)	−1.1 (−2.6, 0.34)	0.14

*Note*: Statistical test uses linear regression, controlling for age, sex, GDS, and education for all. Neuroimaging measures additionally controls for intracranial volume. Descriptive variables are the outcome. The effect size measure is Cohen *d* (except for in the categorical outcomes: sex and microhemorrhages).

Abbreviations: Aβ, amyloid beta; ALMT, African List Memory Test; ANT, Arican Naming Test; *APOE*, apolipoprotein; AQ, Alzheimer's Questionnaire; AVMT, African Visuospatial Memory Test; CI, confidence interval; CSID, Community Screening Instrument for Dementia; GDS, Geriatric Depression Scale; GFAP, glial fibrillary acidic protein; IL, interleukin; NfL, neurofilament light chain; p‐tau, phosphorylated tau; SD, standard deviation; TNF‐α, tumor necrosis factor alpha.

^a^ Results presented as *n* (%) and odds ratio is presented for the effect size measure; logistic regression is used for the statistical test.

^b^ In neuroimaging data interpretation, a higher volume of a structure is generally considered beneficial, whereas atrophy indicates pathology. Regarding biomarkers, a low Aβ42/40 ratio is pathological. Additionally, elevated concentration levels of other plasma biomarkers are also indicative of pathology.

Table 5 presents[Table alz70735-tbl-0003] the sensitivity, specificity,[Fig alz70735-fig-0004] and area under the curve (AUC) for cognitive, neuroimaging, and plasma biomarkers in relation to *APOE* ε4 status. Cognitively, sensitivity ranges from 52% to 80%, specificity from 51% to 75%, and AUC from 65% to 75% (0.53–0.86). Neuroimaging findings indicate that sensitivity varies between 59% and 85%, specificity between 52% and 86%, and AUC between 65% and 84% (0.51–0.94). Plasma biomarkers show that sensitivity ranges from 34% to 75%, specificity from 51% to 93%, and AUC from 64% to 70% (0.52–0.81).

**TABLE 5 alz70735-tbl-0005:** Sensitivity, specificity, and accuracy of cognitive, neuroimaging and plasma biomarkers in distinguishing *APOE* ε4 status.

Variables	Cut‐offs	Sensitivity/specificity	AUC (95% CI)
**Screening and cognitive tests**			
CSID	0.45	0.80/0.54	0.70 (0.59, 0.82)
AQ	0.44	0.80/0.58	0.70 (0.58, 0.81)
ANT	0.42	0.79/0.65	0.74 (0.63, 0.86)
ALMT Trial 1	0.42	0.79/0.50	0.66 (0.54, 0.78)
ALMT Trial 3	0.42	0.85/0.55	0.72 (0.61, 0.83)
ALMT Recall	0.54	0.64/0.73	0.71 (0.60, 0.83)
AVMT Trial 1	0.46	0.72/0.58	0.65 (0.53, 0.77)
AVMT Trial 3	0.43	0.85/0.60	0.75 (0.64, 0.86)
AVMT Recall	0.42	0.82/0.60	0.73 (0.61, 0.84)
Proverb Test	0.55	0.52/0.75	0.67 (0.55, 0.78)
African Card Game Test Wins	0.51	0.67/0.73	0.68 (0.56, 0.80)
GDS	0.46	0.78/0.51	0.65 (0.53, 0.77)
**Neuroimaging** ^a^			
Intracranial volume	0.42	0.74/0.52	0.65 (0.51, 0.80)
Left hippocampal volume	0.50	0.59/0.69	0.67 (0.53, 0.81)
Right hippocampal volume	0.47	0.59/0.76	0.68 (0.54, 0.83)
Left entorhinal cortex volume	0.51	0.63/0.76	0.68 (0.53, 0.82)
Right entorhinal cortex volume	0.40	0.78/0.52	0.66 (0.52, 0.80)
Microhemorrhage	0.40	0.79/0.70	0.78 (0.65, 0.92)
White matter hyperintensity	0.50	0.63/0.72	0.66 (0.52, 0.81)
Mesial temporal atrophy score	0.35	0.85/0.61	0.77 (0.64, 0.89)
Entorhinal cortex atrophy score	0.56	0.69/0.86	0.84 (0.74, 0.94)
**Plasma biomarkers** ^a^			
Aβ_42_	0.50	0.63/0.67	0.67 (0.55, 0.79)
Aβ_40_	0.54	0.53/0.77	0.67 (0.55, 0.79)
Aβ_42/40_	0.49	0.67/0.72	0.70 (0.58, 0.82)
p‐tau_181_	0.59	0.41/0.87	0.65 (0.53, 0.77)
Aβ_42_/p‐tau_181_	0.46	0.75/0.59	0.67 (0.55, 0.79)
NfL	0.48	0.68/0.68	0.70 (0.58, 0.81)
GFAP	0.66	0.34/0.93	0.68 (0.56, 0.79)
TNF‐α	0.45	0.75/0.51	0.64 (0.52, 0.76)
IL‐6	0.44	0.80/0.51	0.67 (0.55, 0.79)
IL‐10	0.49	0.68/0.61	0.66 (0.54, 0.78)

*Note*: Statistical test uses logistic regression, controlling for age, sex, GDS, and education for all. Neuroimaging measures additionally controls for intracranial volume. ε4 carrier status is the outcome.

Abbreviations: Aβ, amyloid beta; ALMT, African List Memory Test; ANT, Arican Naming Test; *APOE*, apolipoprotein; AQ, Alzheimer's Questionnaire; AUC, area under the curve; AVMT, African Visuospatial Memory Test; CI, confidence interval; CSID, Community Screening Instrument for Dementia; GDS, Geriatric Depression Scale; GFAP, glial fibrillary acidic protein; IL, interleukin; NfL, neurofilament light chain; p‐tau, phosphorylated tau; SD, standard deviation; TNF‐α, tumor necrosis factor alpha.

^a^ In neuroimaging data interpretation, a higher volume of a structure is generally considered beneficial, whereas atrophy indicates pathology. Regarding biomarkers, a low Aβ42/40 ratio is pathological. Additionally, elevated concentration levels of other plasma biomarkers are also indicative of pathology.

## 4 DISCUSSION

In the current study, we examined whether *APOE* ε4 is a susceptibility gene for dementia/AD, and we examined its association with cognitive tests, neuroanatomical correlates, and plasma neurodegenerative biomarkers in a sample of old adults in the DRC. This is one of the first exploratory studies to use the *APOE* ε4 allele to explore its genetic susceptibility for dementia/AD in this novel Sub‐Saharan African population. Our results suggested a higher prevalence of the ε3/ε3 genotype in the HC group. Consistent with previous findings, we found a higher frequency of ε4 carriers among the dementia group.^29^

Our first hypothesis, which predicted that *APOE* ε4 will be a significant risk factor for participants with suspected dementia/AD, cognitive deficits, neuroanatomical lesions, and plasma neurodegenerative biomarkers in this DRC sample, was partially supported. As with previous studies,^27^ we found in these preliminary small data that having the *APOE* ε4 allele was a significant genetic risk factor for dementia. We also found that among participants with clinical dementia, there was a significant association between the *APOE* ε4 status and several cognitive tests, including ANT, ALMT trials, and AVMT trials; the latter two sets are tests of memory. Interestingly, cognitively normal participants only demonstrated differences by *APOE* ε4 status on confrontation naming and depression scores. This pattern of results suggests that confrontation naming (ANT test), a measure of language fluency, might be an early marker of cognitive decline among cognitively normal people. These findings are the same as our previous study, which reported the impact of *APOE* ε4 on cognitive deficits.^33^ Our neuroimaging findings in the entire cohort show more atrophy in the mesial temporal lobe and entorhinal cortex among *APOE* ε4 carriers and in participants with clinical[Table alz70735-tbl-0004] dementia.

These findings also highlight the importance of *APOE* ε4 as a genetic risk factor for dementia for old adults in DRC. Although there are few or no studies in Sub‐Saharan Africa that have investigated the effect sizes of various biomarkers and cognitive differences between *APOE* ε4 carriers and non‐carriers in the African population, most of our results align with previous Western findings. Similar to prior studies,^44^ we observed greater cognitive deficits in *APOE* ε4 carriers than in non‐carriers. Additionally, our findings indicate that *APOE* ε4 carriers tend to have higher entorhinal cortex and mesial temporal lobe scores, both of which may contribute to cognitive decline and the progression of dementia.^45^ While other studies have found that abnormal levels of Aβ42 are associated with more rapid cognitive decline and clinical progression to dementia^46^—regardless of *APOE* ε4 status—our study identified a strong positive effect size of GFAP and negative effect sizes of interleukin 10 and tumor necrosis factor alpha on *APOE* ε4 status in participants with dementia. We compared the prevalence of the *APOE* ε4 allele among African populations and found the following: 21.7% among Nigerians (4), 25% among Ugandans (3), 25.4% among South Africans in the Health and Aging in Africa: A Longitudinal Study in South Africa study (2), 29.4% among the Fons of Benin (1), 32.2% among Kenyans (5), 33.3% among Congolese/Zairians, and 38.5% among the Tutsi of Burundi (1). Our results also support the detrimental role of *APOE* ε4 in cognitive deficits, neuroimaging correlates of AD, and two plasma biomarkers (Aβ42/40 and p‐tau181) in old adults with suspected dementia in Sub‐Saharan African populations. These findings are similar to previous studies that found an association between *APOE* ε4 and cognitive functions,^7^, ^8^ temporal lobe atrophy,^15^, ^16^ cortical thinning,^17^, ^18^ and an increase in white matter lesions.^24^, ^25^ As with previous studies, our study found high concentration levels of p‐tau181 and a lower Aβ42/40 ratio, which are associated with AD and dementia.^21^, ^22^ Despite the genetically small sample size, we can tentatively deduce that *APOE* ε4 may be a susceptibility gene for late‐onset sporadic AD in this population. Although this study used cutting‐edge, highly sensitive, and validated plasma tests for AD biomarkers, we were unable to detect differences between controls and suspected dementia cases in the association between *APOE* ε4 and cognitive, neuroimaging, and plasma biomarkers. This is likely due to the heterogeneity of the population regarding the type of dementia, with mixed and non‐AD[Fig alz70735-fig-0005] causes.

This study is in Sub‐Saharan Africa to examine the association between *APOE* ε4 and cognitive,[Table alz70735-tbl-0005] neuroimaging, and plasma biomarkers in a novel sample of old adults in the DRC with and without dementia. Our findings should be interpreted considering several limitations, such as the cross‐sectional nature of the study, low sensitivity, and lack of amyloid positron emission tomography (PET) imaging confirming AD pathology. Future studies should follow a cohort to make longitudinal predictions of the rate of hippocampal atrophy based on baseline plasma biomarkers. Another limitation includes a small sample of participants, which limited the detection of differences that could have been clinically and significantly relevant to finding adequate discriminative strength of the plasma biomarkers. Thus, future studies should replicate these findings with larger sample sizes. Fourth, the screening measures used (CSID and AQ) have not been validated in Sub‐Saharan Africa in general and the DRC in particular. Additionally, this study may have included cases other than amnestic multidomain dementia, increasing the chances of enrolling patients with dementia caused by conditions other than AD and thus not showing significant hippocampal atrophy. Future studies should conduct statistical analyses across all four groups (HCs, mild cognitive impairment, subjective memory complaints, and dementia). Further, only one brain region was analyzed in the current study. Associations with other key regions might have been identified using more powerful imaging techniques such as voxel‐based morphometry. Furthermore, future studies should also aim to replicate our findings using amyloid and tau brain PET or mass spectrometry to measure biomarkers. A major caveat is that our AD biomarkers were determined by Simoa, which is not optimal. The gold standard for core AD biomarker assessment is amyloid and tau brain PET. Head‐to‐head comparisons of amyloid and tau biomarkers have demonstrated the superiority of immunoprecipitation mass spectrometry techniques over Simoa. Thus, continued investigation into racial disparities in AD biomarkers and their relation to AD dementia using these gold standard techniques (e.g., brain amyloid PET, cerebrospinal fluid) is necessary.

## AUTHOR CONTRIBUTIONS

Conceptualization: Jean Ikanga. Data curation: Jean Ikanga. Funding acquisition: Jean Ikanga. Investigation: Jean Ikanga. Methodology: Jean Ikanga. Project administration: Jean Ikanga. Resources: Jean Ikanga. Software: Jean Ikanga. Supervision: Jean Ikanga. Validation: Jean Ikanga. Visualization: Jean Ikanga. Writing—original draft: Jean Ikanga, Saranya Sundaram Patel, and Megan Schwinne. Writing—review & editing: Jean Ikanga, Saranya Sundaram Patel, Megan Schwinne, Caterina Alessandra Obenauf, Emmanuel Epenge, Guy Gikelekele, Nathan Tshengele, Immaculee Kavugho, Samuel Mampunza, Lelo Mananga, Charlotte E. Teunissen, Julio C. Rojas, Brandon Chan, Argentina Lario Lago, Adam L. Boxer, Andreas Jeromin, Alden L. Gross, Alvaro Alonso. Formal analysis: Megan Schwinne.

## CONFLICT OF INTEREST STATEMENT

AJ was employed by ALZpath, Inc. The remaining authors declare that the research was conducted in the absence of any commercial or financial relationships that could be construed as a potential conflict of interest. Author disclosures are available in the supporting information.

## ETHICS STATEMENT

The studies involving humans were approved by University of Kinshasa and Emory University. The studies were conducted in accordance with the local legislation and institutional requirements. The participants provided their written informed consent to participate in this study

## Supporting information



Supporting information

## Data Availability

The raw data supporting the conclusions of this article will be made available by the authors, without undue reservation.
